# Complete bladder duplication presenting in adulthood: A case report on reconstructive technique and reflections on translational urology in refugees

**DOI:** 10.3389/fruro.2023.1080410

**Published:** 2023-02-21

**Authors:** David Abramowitz, Wietse Claeys, Caroline Jamaer, Camille Berquin, Piet Hoebeke, Anne-Françoise Spinoit

**Affiliations:** ^1^ Department of Urology, University at Buffalo Jacobs School of Medicine, Buffalo, NY, United States; ^2^ Department of Urology, Ghent University Hospital, eUrogen Accredited Center European Reference Network (ERN), Ghent, Belgium

**Keywords:** bladder exstrophy, transition, socio-politic background, duplication, reconstruction

## Abstract

**Introduction and aim of study:**

The bladder-Exstrophy-Epispadias (BEEC) complex is a spectrum of congenital malformations with many variations. A never operated political refugee with BEEC was referred to our center for management upon arrival in Europe. Our aim is to report the technique and outcomes on a never operated on BEEC adult, highlighting the importance of transitional urologic care for congenital malformations in adult patients.

**Materials and methods:**

A 27-year old female patient was referred to our center for complete incontinence since birth by the General practitioner from the refugee center who suspected BEEC. Upon further investigation, an exstrophic bladder with blind ending ureteral orifices and a urethral meatus caudal to the exstrophic bladder plate were highlighted. A second non-exstrophic bladder with two orthotopic ureters was demonstrated, thereby a bladder duplication in the sagittal plane was diagnosed, presenting a wide-open bladder neck and a 7 cm pubic diastasis, causing the incontinence she was initially referred for. With the patient in a supine position, laparotomy incision was done with excision of the umbilical scar. The exstrophic bladder plate is dissected caudally. As it presents good detrusor quality, decision is taken to use it as a ventral inlay to augment the non-exstrophic bladder. A Mitchell-type bladder neck reconstruction is performed with a classical fascia sling wrapped around the bladder neck to increase the continence mechanism given the very wide pubic diastasis. Given the risk for hyper-continence, interposition of a continent Mitrofanoff-type vesicostomy is additionally realized. Genital reconstruction is achieved.

**Results:**

Over 1 year post operatively, the patient is completely dry, can holp up to 250ml between catheterization she performs five times per day and once at night. No post-operative complications were observed.

**Conclusion:**

The case of one adult patient with a rare urological condition like bladder exstrophy with duplication is presented, illustrating challenges political refugees referred to Europe implicates in terms of surgery regarding congenital malformations in adult patients. A multidisciplinary approach is highly important, demonstrating the importance of transitional care.

## Introduction

Bladder duplication is a rare congenital anomaly with relatively few case reports available in the literature (1–6). In most of these, the phenomenon occurs along with other congenital malformations. Abrahamson initially described bladder duplications in 1961 into two distinctions - complete or incomplete duplication (7). The great majority of these anomalies are corrected in early childhood. Unfortunately, as regional instabilities and cultural condemnation continue to exist in parts of the world, immigrants and refugees may present in the arrival countries for treatment of these anomalies at an older age. Here we describe the case of an adult female individual presenting at our center with an isolated bladder duplication in the sagittal plane that was never treated before.

## Case presentation

**Figure 1:**
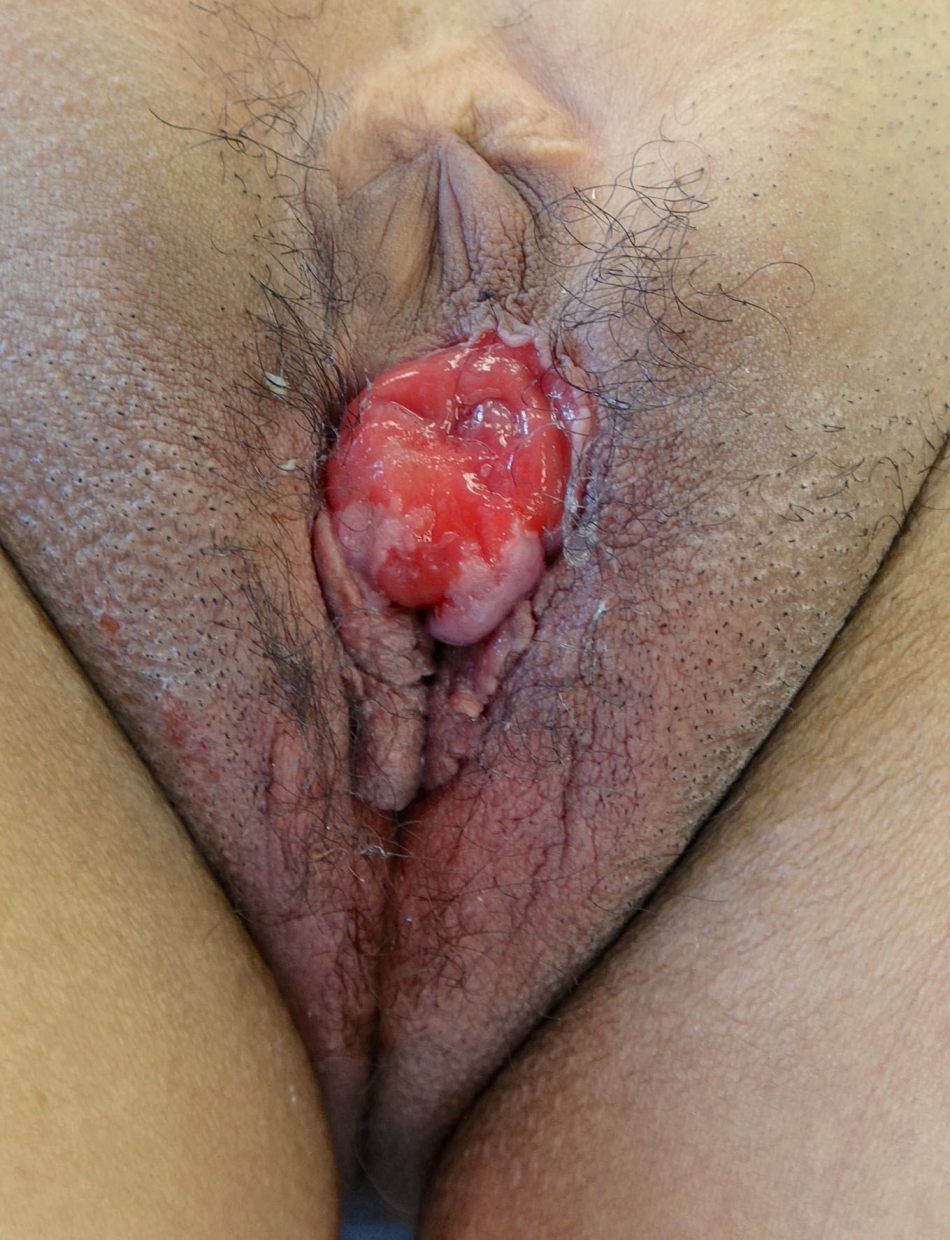
The exstrophic bladder is visible between the umbilical scar, located very caudally on the abdomen, en the introitus with the labia maiora.

Our patient is a 27-year-old female who presented to our tertiary referral center for complaints of lifetime total urinary incontinence. She emigrated from Syria six months prior. The general practitioner from a refugee center referred her with suspicion of open bladder exstrophy (**Figure 1**). She reported no history of urinary tract infections. Gynecologically, she reported regular menses and had never been sexually active. She stated her parents had prohibited her any form of social interaction and kept her inside out of shame for her condition. Only recently, she managed to escape during socio-political instabilities in the country.

**Figure 2:**
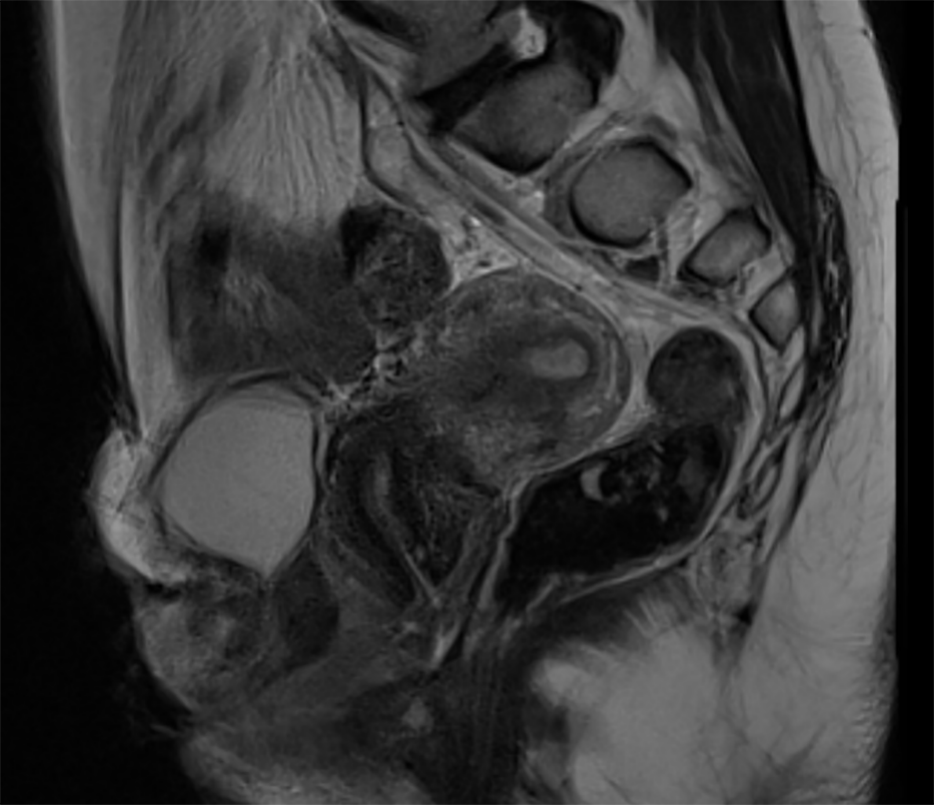
MRI of the abdomen/pelvis of the patient. Sagittal view from the patient. Anteriorly the non-exstrophic bladder is visible, uterus and rectum are clearly visible.

Initial exam under general anesthesia included a cystoscopy and vaginoscopy and showed an exstrophic bladder with blind ending ureteral orifices. A second, non-exstrophic bladder was identified posterior to the exstrophic one with a broad open bladder neck and an estimated capacity of 150ml. This would be considered a complete bladder duplication in the sagittal plane. Vaginoscopy and subsequent hysteroscopy showed a narrow vaginal canal with a normal cervix and an uterus without any connections to the urinary tract. Normal anorectal anatomy was visualized and palpated as well. CT imaging showed a normal upper urinary tract with normally formed bilateral kidneys, and an arcuate uterus. Additional voiding cystourethrogram showed non-refluxing ureters. Karyotyping was performed and showed a 46XX pattern. Biochemical work-up showed a normal kidney function and normal hormone levels. She desired to be continent and to have a more normal appearing anatomy. MRI of the pelvic area was further performed to guide the reconstruction but did not reveal any new abnormalities (**Figure 2**). Pre-operative evaluation was performed in the transitional clinic setting, with cooperation from gynecologists, psychologists, nurse specialists, social workers, and in this specific case, orthopedics.

**Figure 3:**
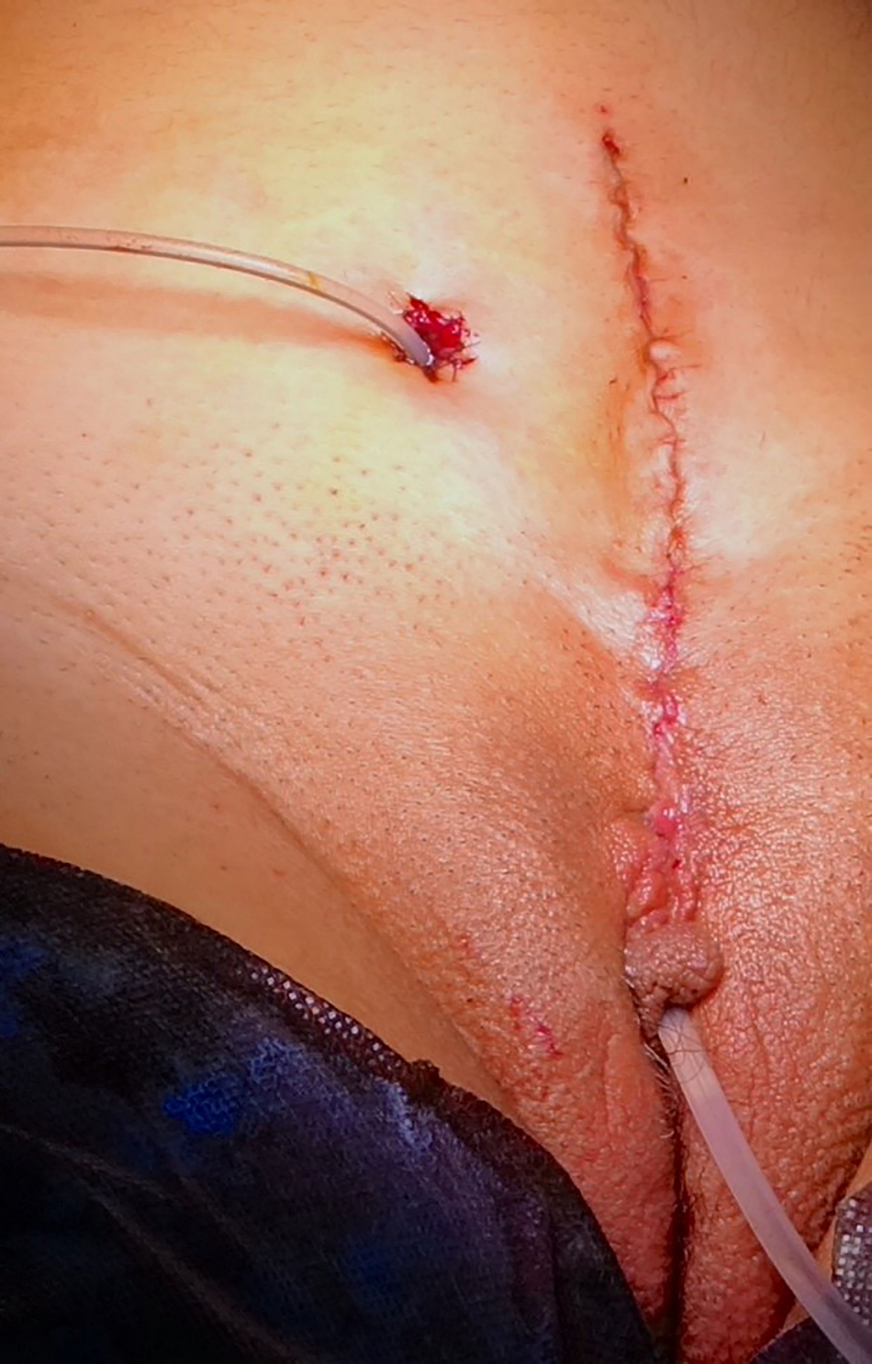
Post-operative image, with a transurethral catheter coming out caudally after reconstruction and on the left the catheter placed though the appendico-vesicostomy.

Reconstruction in this case was performed by two experienced urologists specialized in pediatric and reconstructive urology. Our operative technique was as follows. A Foley catheter was placed in the meatus of the posterior internal bladder for identification. An infraumbilical laparotomy incision was made and continued around the exstrophic bladder. The two bladders were dissected free from each other and the exstrophic bladder was used to augment the intrapelvic bladder in a ventral onlay fashion. Upon dissection of the non-exstrophic bladder plate, we found that the vascularization mainly stemmed from the median bladder neck. Therefore, a pedicle from this bladder neck to the exstrophic bladder plate was preserved, and a pivoting plane between the exstrophic and non-exstrophic bladder was created. The opening of the non-exstrophic bladder anteriorly revealed that the ureteric ridge was sufficiently apart from the bladder neck so that a Mitchell type bladder neck reconstruction could be performed. Redundant bladder neck tissue was used to elongate the urethra. A plane between the urethra and anterior vaginal wall was then created for placement of a fascial sling. A 2x8cm portion of rectus fascia was deemed long enough and harvested with the right lateral aspect of the graft staying attached to the rectus sheath. The sling was wrapped around the proximal urethra and sutured to the left side of the diastatic pubic bone after appropriate tensioning. The exstrophic bladder plate was then augmented onto the ventral aspect of the non-exstrophic bladder by turning the caudal edge of the plate towards the cranial aspect of the opened non-exstrophic bladder. In this way the vascular pedicle supporting this plate comes caudal and anterior. Due to it not being clear whether the patient’s newly augmented single bladder would be able to empty on its own, a continent catheterizable Mitrofanoff Type appendicovesicostomy channel was created. Since the patient had stable gait pattern, the diastatic pubic bone was not closed. After closure of the abdominal wall, external genital reconstruction was performed to restore a normal appearing vulvovaginal complex. The clitoris could not be completely reconstructed due to insufficient remaining tissue. At the end of the surgery, a transurethral as well as a trans appendiceal catheter were left in place (**Figure 3**). The patient was admitted for ten days, until full maturation of both channels was achieved. Urinary function remained stable. Upon removal of the transurethral catheter, we found that the patient was unable to void spontaneously. Therefore, clean intermittent self-catheterization of the appendiceal channel was taught, so that the patient could still empty her bladder while cycling the bladder.

Postoperative follow-up was performed at our tertiary clinic by one of the two surgeons. Upon first postoperative visit, the patient did well and experienced no major complications according to the Clavien-Dindo grading system. However, the patient remained unable to void spontaneously and remained using her catheterizable channel. Upon repeat visit four months after surgery, the patient complained of intermittent urgency and urinary loss from the urethrostomy. A trial with oxybutynin 5mg three times per day could completely solve the complaint of urgency, but the urethrostomy continued to leak upon Valsalva and other movements raising intra-abdominal pressure. This was likely due to insufficient coaptation capability of the reconstructed bladder wall. We thus opted for the use of an ACE stopper in between self-catheterizations. At one year follow-up, the reconstructed bladder can hold 250ml of urine and the patient can maintain a schedule of self-catheterization of five times per day and once at night. The kidney function remains stable and the ureters did not show reflux on repeat cystography. She had no further complications and is satisfied with the reconstruction. Further urodynamic investigation was not deemed necessary as the bladder function remained acceptable to date.

## Discussion

This was a particularly unique case of complete bladder duplication for a number of reasons. There are approximately 70 described cases of bladder duplication in the literature and most of them have concomitant congenital abnormalities of adjacent embryologic structures as well (1–6). This being an isolated duplication of the lower urinary tract made its reconstruction less complicated and therefore more likely to be successful. Given patient had never voided, her voiding function could not be evaluated. Moreover, she refused urodynamic evaluation for cultural reasons. We opted for bladder neck reconstruction with a sling, and given the risk of hyper-continence, a catheterizable channel (Mitrofanoff) was chosen as option if she could not void postoperatively. Of course, multiple types of continence reconstruction are possible in a case like this. As this patient wished to remain in our country for the foreseeable future, the availability of sterile materials such as single-use catheters was not deemed to be a problem. In other settings where the both recourses to care and to specific and medically safe materials would be more of an issue, a less resource demanding technique, such as a Mainz-II-pouch may be more suitable, even if we prefer not to use this kind of pouch in young patients given the neoplastic risk.

Presentation of this type of isolated uncorrected bladder duplication in adulthood does not seem to have been described in the literature prior to this report. Most congenital urologic anomalies are repaired in the first few years of life. As children with reconstructions age and go through puberty, however, they can “grow out” of their repair and require subsequent reconstructions up to early adulthood (8). Transitional care is of utmost importance and an evolving area of expertise in urology (9). Patient was taken care of in a tertiary center completely adapted for this rare condition, as the transition urology clinic involves a team of both pediatric and adult urologists, gynecologists, nurse specialists, nephrologists, psychologists and endocrinologists.

As our world population continues to increase, so does the migration of people from one region to another. Interstate as well as domestic conflicts may lead to abrupt waves of migration from countries with less developed healthcare systems to countries with more availability to medical resources. According to the United Nations High Commissioner for Refugees (UNHCR), there were 82.4 million displaced refugees at the end of 2020 (10). Due to both ethnic and religious differences in acceptance towards medical conditions in various parts of the world, receiving physicians may be faced with unique presentations of a wide range of medical and surgical issues in later stages of both the disease and life of the patient. The involvement of multidisciplinary teams including both pediatric and adult urologists will improve the transitional care process of patients with these conditions and aid in understanding the major hurdles faced when living with complicated congenital urological issues.

## Conclusion

Here we presented a case of an isolated bladder duplication with an exstrophic bladder with total lifetime incontinence that was not treated until adulthood. She had successful reconstruction of her bladder and external genitalia and is now socially continent through the augmentation of her non-exstrophic bladder and a catheterizable channel. However, she remained in need for an ACE stopper to provide this continence. Along with the increase in refugees coming from regions with subpar medical care, these unique presentations of congenital anatomic abnormalities in adulthood may become more frequent in the Western world. No single technique will be suitable to all. Therefore, it is of utmost importance that reconstructions like this take place in highly specialized centers with multidisciplinary care.

## Data availability statement

The raw data supporting the conclusions of this article will be made available by the authors, without undue reservation.

## Ethics statement

Ethical review and approval was not required for the study on human participants in accordance with the local legislation and institutional requirements. The patients/participants provided their written informed consent to participate in this study. Written informed consent was obtained from the individual(s) for the publication of any potentially identifiable images or data included in this article.

## Author contributions

DA: Literature research, manuscript writing and editing. WC: critical review, literature research and manuscript writing. CJ: Manuscript writing, patient care, concept realization. CB: Patient care, manuscript editing, concept realization. PH: Surgery, Revision of manuscript. A-FS: Surgery, patient care, concept realization, manuscript writing and editing. All authors contributed to the article and approved the submitted version.
